# Fishy business in Seattle: Salmon mislabeling fraud in sushi restaurants vs grocery stores

**DOI:** 10.1371/journal.pone.0311522

**Published:** 2024-11-06

**Authors:** Jewel L. Garcia, Yennifer A. Gaspar, Angelique Djekoundade, Mhicca Dalere, Asmaa A. Al-awadi, Marjolene Allossogbe, Thania C. P. Allossogbe, Itzel S. Aparicio, Hannah N. Buller, Hera Beatrice F. Cadelina, Isabella K. Camarillo, Kayla Case, Abigail E. Dean, Sara M. Dean, Jordyn F. DeJong, Elizabeth Delgado, Renske J. Dupar, Emma N. Ely, Mia C. Ewing, Delina N. Filli, Spencer E. Fleming, Mackenzie R. Garrett, Blair P. Graves, Marie M. Hafez, Weston P. Hanson, Alexander D. Heller, Anthony J. Hernandez, Elizabeth K. Horton, Ellie G. Jancola, Lauryn A. Keith, Madison J. Knoke, Jared D. Larkin, Andre’ G. Marineau, Fabiola Martin-Ortiz, Olivia L. Mayer, Yolanda M. Mendoza, Peter V. Nalivayko, Nguyen Nguyen, Eloisa T. Nguyen, Henry Nguyen, Griffin L. Ovenell, Lay G. Paw, Spencer R. Raymond, Janetta J. Redzic, Madelyn T. Rice, Ashlie T. Rodrigo, Jonathan M. Savell, Ben R. Sheirbon, Dulce S. Torres, Kalena A. Warrick, Eric S. Long, Timothy A. Nelson, Tracie Delgado

**Affiliations:** Department of Biology, Seattle Pacific University, Seattle, Washington, United States of America; University of Messina, ITALY

## Abstract

Salmon is the most commonly consumed finfish in the United States of America (USA), and the mislabeling of salmon is a widespread problem. Washington State is a global supplier of wild-caught Pacific salmon and local salmon mislabeling results in substantial economic, ecological, and cultural impacts. Previous studies in Washington State identified high levels of mislabeled salmon in both markets and restaurants, resulting in local legislation being passed that requires proper labeling of salmon products, including identifying it as wild-caught or farm-raised. To investigate whether recent legislative efforts reduced salmon fraud rates, we acquired and genetically barcoded salmon samples from 67 grocery stores and 52 sushi restaurants in Seattle, Washington. DNA from each salmon sample was isolated and the cytochrome c oxidase gene was sequenced to identify the fish species. Our study, conducted from 2022–2023, revealed 18% of salmon samples from both grocery stores and sushi restaurants were mislabeled. While most samples were acquired during the fall months when wild salmon is in season, we still observed a high salmon mislabeling rate. Unlike grocery stores, Seattle sushi restaurants often sold farmed salmon mislabeled as wild salmon. Specifically, substitutions of vendor-claimed wild salmon with farmed salmon occurred in 32.3% of sushi restaurant samples compared to 0% of grocery store samples. Additionally, occurrences of wild salmon being substituted with another salmon species (wild or farmed) occurred in 38.7% of sushi restaurant samples compared to 11.1% of grocery store samples. All salmon substitutions in sushi restaurants harmed the customer financially as they were given a cheaper market-priced fish. In grocery stores, however, we did not detect significant economic loss to customers due to salmon mislabeling. Taken together, it is important to continue to develop and enforce legislation in Washington State that prevents salmon fraud and promotes ecologically sustainable fishing practices.

## Introduction

In recent decades, the global scale of fisheries declines has become increasingly evident [1–3]. Factors that contribute to large-scale fisheries declines include increased fishing effort [4], technological advancement which increases fishing pressure [5], and dishonest reporting of catch data which complicates management efforts [6]. More recently, the problem of poorly labeled and mislabeled seafood has received increasing attention, as labeling inconsistencies obscure provenance and undermine effective fisheries management efforts [7, 8]. Multiple seafood mislabeling studies have been conducted in domestic United States of America (USA) cities [9–18] and in countries outside the USA [12, 19–31]. Using DNA barcoding of the highly conserved mitochondrial cytochrome c oxidase I (COI) gene in fish, it is possible to identify the genus or species of seafood products from a small tissue sample and determine if it is mislabeled [32].

A 2013 Oceana seafood fraud study in the USA found 33% of seafood samples were mislabeled [17]. Of the most common sampled fish species, red snapper had the highest mislabeling rate at 87% [17]. Furthermore, a meta-analysis of 28 seafood products estimated an 8% global seafood mislabeling rate [31]. In the USA, salmon has the highest overall commercial value [33] and the highest consumption rate of all finfish [8], therefore salmon mislabeling rates are of huge interest to the American public. Research by Oceana found a 7% salmon mislabeling rate in the USA during peak wild salmon season [17] and a 43% mislabeling rate during the off-season months [18]. Furthermore, a meta-analysis study found Americans are more likely to eat a mislabeled salmon than any other finfish due to its high consumption rate in the USA [8].

Washington State is a major supplier of wild Pacific salmon. In Washington State, there are five native species of anadromous Pacific salmon: Chinook (*Onchorhyncus tshawytscha*), chum (*Onchorhyncus keta*), coho (*Onchorhyncus kisutch*), sockeye (*Onchorhyncus nerka*), and pink (*Onchorhyncus gorbuscha*) [34]. Additionally, there are two native species of iteroparous anadromous trout within the same genus: coastal cutthroat (*Onchorhyncus clarkii clarkii*) and steelhead trout (*Onchorhyncus mykiss*) [34]. Not native to the Pacific Ocean, nearly all global farmed salmon are Atlantic salmon (*Salmo salar*) [35]. Historically abundant throughout the northeastern USA, wild Atlantic salmon have been reduced to a single, endangered, distinct population segment in the Gulf of Maine [36]. Thus, commercial fishing of wild Atlantic salmon is prohibited within the USA, and all Atlantic salmon sold domestically are farmed [37]. Because Washington State supplies a significant amount of wild-caught Pacific salmon in the USA, mislabeling of salmon in Washington State could have substantial economic, ecological, and cultural impacts.

Salmon have been an integral part of the Pacific Northwest and its people for millennia. Seattle, Washington (WA), is on the migratory pathways of salmon returning to the south and central Puget Sound rivers to spawn. Seattle’s waters include the Salish Sea, an inland sea of the Pacific Ocean that spans Washington State (USA) to British Columbia, Canada. The city of Seattle is deeply rooted in the culture and history of its Native Suquamish and Duwamish People, and salmon have sustained generations of Native Americans who have named themselves as the “*Salmon People*.” For over 6,000 years, the Coast Salish native peoples have inhabited the Pacific Northwest and have depended on salmon as a critical resource, as well as a means to celebrate and unify their peoples along the coast [38]. Salmon has since become a cultural icon and heritage for people living in Seattle, with salmon found in most markets, displayed as art around the city, and a common food source.

Salmon is an important part of Washington’s economy and local businesses. It is estimated that ~1.67 million Washington State adults consumed salmon in 2010 and ~2.9 million adults will consume salmon in 2030 [39]. In Washington State waters, ~17 million pounds of salmon were harvested in 2022, earning ~$26 million in revenue [40]. As of 2022, nine populations of Pacific salmon are still endangered in Washington State [41]. In partnership with Native American governments, Washington State has enacted numerous salmon restoration efforts such as decreasing the wild salmon being harvested, improving salmon hatcheries, and restoring and clearing estuaries and waterways [41].

Historically, salmon mislabeling has been observed in Washington State [9, 17], but recent legislative changes have aimed to reduce mislabeling fraud. From 2009–2011, a study conducted in Washington State found 20 out of 99 (20%) of salmon samples in grocery stores and restaurants combined were mislabeled [9]. This study also found wild salmon was substituted with farmed salmon in 2% of grocery store samples and 24% of sushi restaurant samples [9]. In 2012, a 60 salmon sample study in Seattle, WA. revealed 2% of salmon samples in grocery stores and restaurants combined were mislabeled [17]. The decline in salmon fraud in Seattle and the surrounding Puget Sound area was likely attributed to the 2011 federal prison sentence of a salmon distributor out of Bellingham, WA who intentionally sold over 160,072 pounds of cheaper market-priced coho salmon as higher market-priced Chinook salmon, with estimated fraudulent profits of $1.3 million [42]. In 2013, as a result of these salmon fraud revelations, Washington State passed House Bill (HB) 1200 which made it unlawful to knowingly sell any fresh, frozen, or processed fish or shellfish products without identifying its common name, so a buyer can make an informed purchasing decision [43]. HB 1200 also made it unlawful for any retail salmon (fresh, frozen, or processed) to be sold without identifying it as wild-caught Pacific salmon or farm-raised Atlantic salmon. People who violated HB 1200 would risk jail time as well as be subject to financial penalties.

There has been a growing ecological concern with allowing farmed Atlantic salmon along the Pacific coast, in part due to public fear of resource competition and interbreeding between wild Pacific salmon and escaped farmed Atlantic salmon [44]. In 2017, a farmed salmon net pen collapsed and at least 243,000 farmed Atlantic salmon escaped near Cypress Island, WA into the Puget Sound [45]. In 2022, resulting public pressure led Washington State to join other western USA states (i.e. California, Oregon, and Alaska) in prohibiting commercial finfish farming in state waters, including farmed Atlantic salmon, although terrestrially-based fish farming remains legal [46].

To investigate whether recent legislative efforts have reduced salmon mislabeling rates compared to 12 years ago, we acquired and tested 119 salmon samples from various grocery stores and sushi restaurants in Seattle, WA. When salmon samples were mislabeled, we identified which species were used as substitutes. Our study is unique for a few reasons. First, we sourced all our samples within the boundaries of one major USA city, while most studies we have seen acquired samples far beyond city limits, over multiple state lines, or various countries [9, 10, 12, 16, 19–22, 25–28, 30]. Second, our study has one of the largest regional salmon sample sets (n = 119). Most other studies tested multiple seafood products as part of the same study, not just salmon, and therefore had far fewer samples per fish product tested [10, 15, 19, 20, 23, 24, 26, 28, 29]. Third, we purposely acquired one salmon sample per vendor to facilitate broader sample coverage throughout the city of Seattle. Fourth, since we focused our study entirely on salmon, not only were we able to determine overall salmon mislabeling rates, we also were able to identify various categories of salmon substitutions. Overall, the results of this study will, 1) reveal how compliant Seattle grocery stores and sushi restaurants are with Washington State seafood laws, 2) impact not just Seattle and Washington State, but also the rest of the country as Washington State is a major source of Pacific salmon in the USA, and 3) foster public awareness of salmon mislabeling rates and their role in wild salmon sustainability.

## Materials and methods

### Salmonid classification

Salmonid species examined in this study included five native Pacific salmon species (i.e., Chinook, sockeye, chum, coho, and pink), two native trout (i.e., steelhead and coastal cutthroat) and Atlantic salmon. We collected samples purportedly from farmed Atlantic salmon and the three most commonly marketed wild Pacific salmon species: sockeye, coho, and Chinook (advertised as “king” salmon). Genetically, Pacific salmonids in genus *Oncorhyncus* comprise a monophyletic clade, with Atlantic salmon more distantly related [47, 48].

### Salmon sample collection

**Fig 1:**
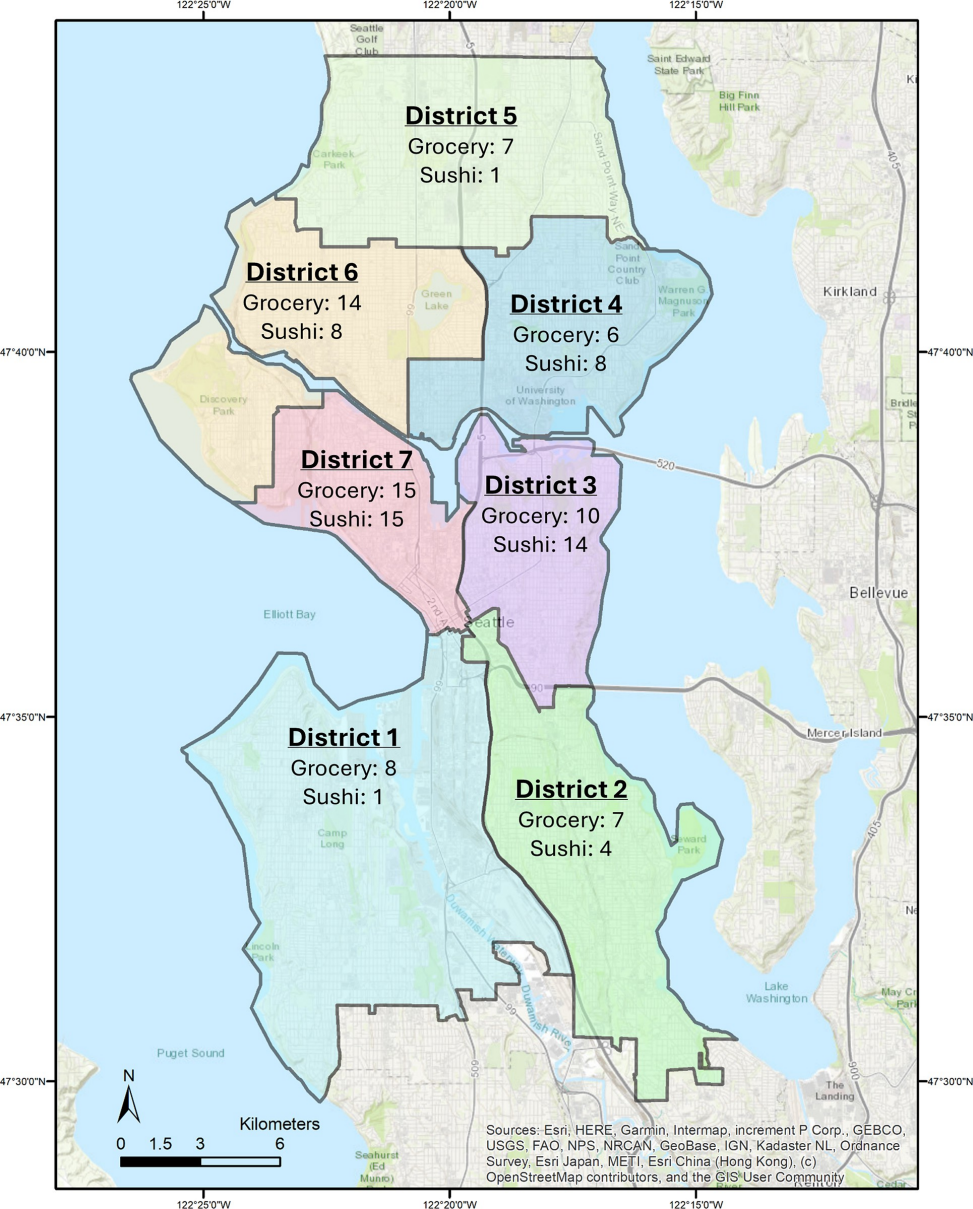
Map of the 7 city council districts in Seattle, WA. Salmon sample numbers obtained from grocery stores or sushi restaurants for each city council district in Seattle, WA. are displayed. Seattle basemap is the intellectual property of Esri (Redlands, California) and is used herein under license and permission by Esri. Copyright @ 2013 Esri. All rights reserved. Map sources include Esri, HERE, Garmin, Intermap, increment P Corp., GEBCO, USGS, FAO, NPS, NRCAN, GeoBase, IGN, Kadaster NL, Ordnance Survey, Esri Japan, METI, Esri China (Hong Kong), (c) OpenStreetMap contributors, US Census Bureau, and the GIS User Community.

We collected and analyzed fresh or previously frozen salmon fillet tissue samples from 67 different grocery stores and 52 different sushi restaurants within all seven congressional districts of Seattle, WA (Fig 1). Samples were collected in fall 2022 (n = 31, sushi), winter 2023 (n = 15, sushi), spring 2023 (n = 6, sushi), and fall 2023 (n = 67, grocery) (S1 and S2 Tables). The salmon species name was documented after verbal declaration by vendors using common market names [Chinook (king) salmon, sockeye salmon, coho salmon, or Atlantic salmon] (S1 and S2 Tables). If the vendor did not know what species of wild Pacific salmon a particular sample was, the sample was then documented as “wild caught (unknown species).” Clean gloves were used to transfer each salmon tissue sample into labeled 15 mL tubes with unique identification numbers. Only one salmon sample was acquired from each establishment to allow larger and broader sample coverage of Seattle grocery stores and sushi restaurants. The 67 grocery store samples included 29 national chain grocery stores, 16 local chain grocery stores, and 22 independently run grocery stores. Specific vendor names are not disclosed herein. Samples were stored temporarily at 4°C for up to two days before being moved to long-term storage at -20°C.

### Salmon DNA isolation & quantification

A 25 mg piece of salmon tissue was extracted from the interior of each salmon sample using a new razor blade and fresh gloves to minimize DNA cross contamination. Salmon tissue samples were lysed overnight and its DNA was isolated using the Qiagen “DNeasy Blood and Tissue” kit (# 69504) according to the manufacturer’s specifications. DNA concentration was quantified at 260 nm using the Thermo Scientific “NanoDrop One” instrument.

### Polymerase chain reaction (PCR) amplification of the cytochrome c oxidase (COI) gene

After DNA isolation, PCR was used to amplify the mitochondrial COI gene, which has been shown by several studies to be very useful in genetically identifying various fish by genus or species [9, 12, 15, 20, 25, 32, 49, 50]. PCR amplification was performed using the Promega “GoTaq Flexi DNA Polymerase PCR” assay kit (#M8295) and Promega “PCR nucleotide mix” (#C1141). Fish DNA barcoding was achieved by ordering previously published primers from Integrated DNA Technologies, which amplify a ~652 bp sequence in the mitochondrial COI gene: Fish F1 primer (5’-TCAACCAACCACAAAGACATTGGCAC-3’) and Fish R1 primer (5’-TAGACTTCTGGGTGGCCAAAGAATCA-3’) [32]. The PCR reaction included 250 ng of DNA, 2.5 U DNA Polymerase, 2.5 mM MgCl_2_, 1x buffer, 0.2 mM dNTP, 25 nM Fish F1 primer, 25 nM Fish R1 primer, and nuclease-free water to a final volume of 50 μL. PCR amplification was performed in the Biorad T100 thermal cycler at the following specification: warm up (96°C for 5 min), 42 cycles of PCR (94°C for 1 min, 51°C for 45 s, and 72°C for 1 min), final extension (72°C for 5 min), and final hold (12°C). A PCR negative control (non-template blank) was included and resulted in no amplification. After PCR amplification of the COI gene, each PCR sample was cleaned using the Promega “Wizard SV Gel and PCR Clean-Up System” kit (#A9281) according to the manufacturer’s specifications. Purified PCR DNA was quantified at 260 nm using the Thermo Scientific “NanoDrop One” instrument. Next, PCR amplification of the ~652 bp COI gene was confirmed via gel electrophoresis on a 1.2% agarose gel after mixing 5 μL PCR purified DNA, 5 μl nuclease-free water, and 2 μL of 6x loading buffer.

### DNA barcoding: Sanger DNA sequencing and analysis

All COI amplified PCR products were prepared for sequencing by mixing 50 ng of PCR amplified DNA, 3 μL of 3 μM Fish F1 primer, and nuclease-free water to a final volume of 8 μL. Samples were then submitted to the Fred Hutchinson Cancer Center Genomics and Bioinformatics Core where Sanger DNA sequencing was performed. Sequencing files (S1 Data) were analyzed using the FinchTV chromatogram viewer and the FASTA DNA sequences were exported. The FASTA DNA nucleotide sequences were aligned using the National Center for Biotechnology Information Basic Local Alignment Search Tool nucleotide (BLASTn) search website (https://blast.ncbi.nlm.nih.gov/Blast.cgi). The top and closets matching fish DNA sequence in the BLASTn database was used to determine the identity of each sample. Any instance where the “vendor-claimed” salmon species identity did not match the “BLASTn” DNA sequence identity, the fish sample was categorized as mislabeled.

### Salmon mislabeling rates

Salmon mislabeling results were grouped into four categories: 1) wild to farmed substitution (Pacific salmon species is substituted with Atlantic salmon), 2) wild salmon to another wild salmon substitution (Pacific salmon species is substituted with a different Pacific salmon species), 3) farmed to wild (Atlantic salmon is substituted with a wild Pacific salmon species), and 4) salmon to non-salmon (any salmon species is substituted with a fish that is not salmon). Grocery stores sold 54 samples as wild Pacific salmon and 13 samples as farmed Atlantic salmon (total grocery store n = 67). Sushi restaurants sold 31 samples as wild Pacific salmon and 21 samples as farmed Atlantic salmon (total sushi restaurant n = 52). Wild salmon substitution rates were determined by analyzing only the vendor-claimed wild salmon samples, and excluding any vendor-claimed farmed salmon samples, in grocery store samples (n = 54) and sushi restaurant samples (n = 31).

### Salmon substitution consumer gain vs loss analysis

When considering the question of who benefits during substitutions, customers or vendors, we determined the average grocery store market price ($/pound) of the four salmon species collected during our study (Chinook, coho, sockeye, and Atlantic salmon). When the vendor claimed it was “wild” salmon but did not know what kind of wild salmon it was, we conservatively priced it as coho salmon since it was the cheapest wild salmon in our analysis. In the case where farmed Atlantic salmon was substituted with steelhead trout (*O*. *mykiss*), also known as rainbow trout, the price of steelhead trout was not available from the store it was purchased from. Instead, we priced steelhead trout by taking the average market price of steelhead across five different Seattle grocery stores. Grocery store salmon species prices were used to determine customer gain or loss in both grocery stores and sushi restaurants, as sushi restaurants displayed a larger price variability because of multiple factors, such as 1) how many sushi pieces were in a roll, 2) some salmon sushi samples were priced as nigiri (raw salmon over rice), or 3) pricing varied based on normal vs “happy hour” discounted pricing. Negative (-$/pound) cost analysis demonstrates a net customer loss per event as the customer is given a cheaper market-priced fish than paid for. Positive (+$/pound) cost analysis demonstrates a net customer gain per event as the customer is given a more expensive market-priced fish than paid for. To determine who benefits during substitutions, we analyzed mislabeled samples in grocery stores and sushi restaurants. Any events where the customer gained (+$/pound) due to substitution was labeled as “customer benefits.” Any events where the customer lost (-$/pound) due to substitution was labeled as “vendor benefits.”

### Statistical analyses

Using Fisher’s Exact Test (R Studio 2023.12.0 Build 369) on a series of 2X2 contingency tables (correct vs. incorrect identification and grocery store vs sushi restaurants), we determined if error rates varied between grocery stores and sushi restaurants when comparing 1) overall error rates, 2) misidentification of farmed fish as wild-caught, 3) all misidentification of putative wild-caught fish species, and 4) economic impact on the consumer (i.e., did the error benefit or harm the consumer). The p-values from these tests were corrected by hand using the Dunn-Sidak method [51] to control inflating type I error probabilities. For all analyses, α was set to 0.05. We also compared the distribution of financial effects on the consumer (benefits or harms of misidentification) between grocery stores and sushi restaurants by conducting a Mann-Whitney U test (IBM SPSS Statistics v. 29.0.1.0). The reported U value was found as U’ based on SPSS output and following the method outlined by Zar [51]. The reported p-value was the exact, rather than asymptotic, as calculated in SPSS.

## Results

### Salmon mislabeling rates in Seattle grocery stores and sushi restaurants

**Fig 2:**
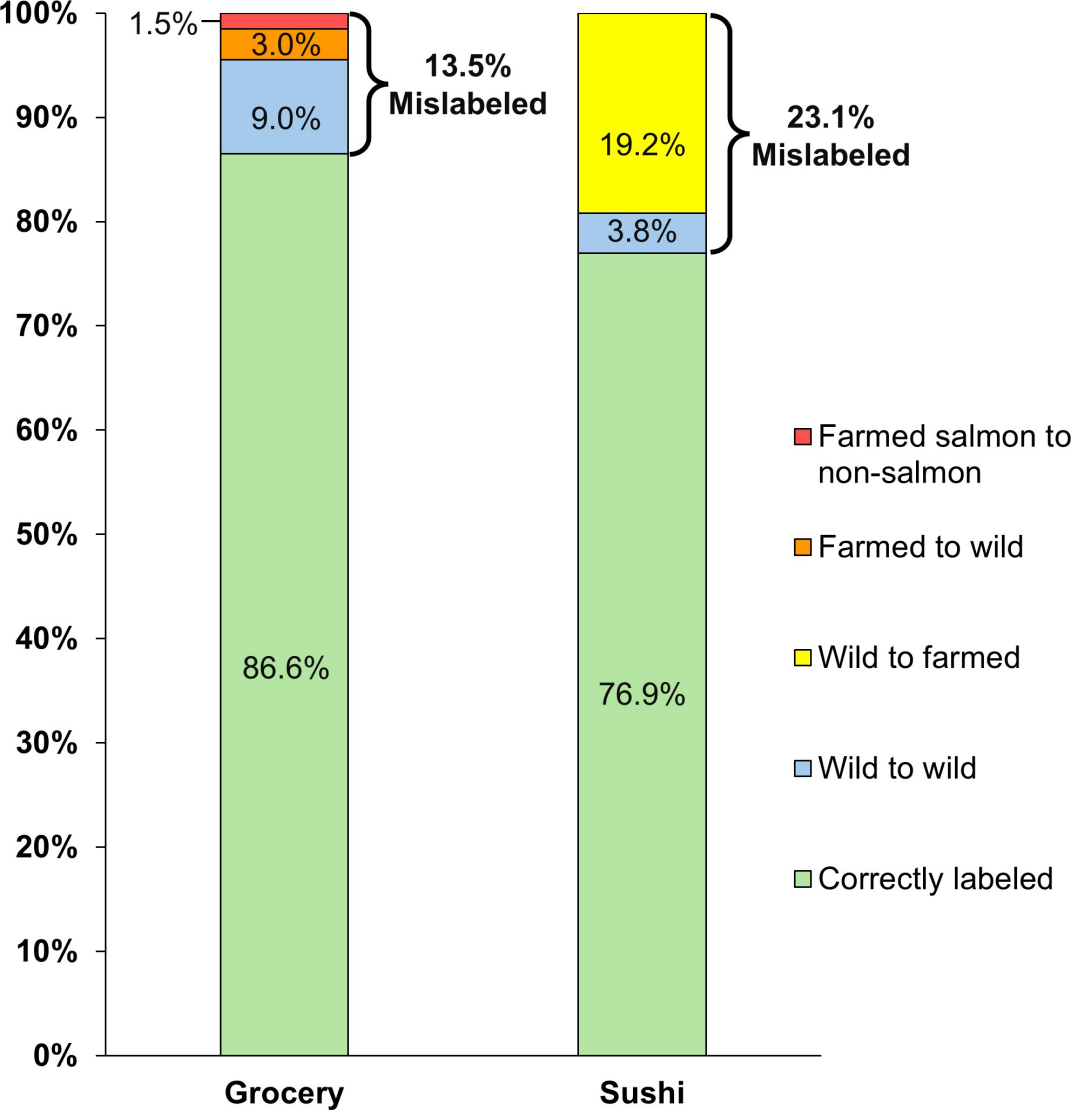
Salmon mislabeling rates in grocery stores vs sushi restaurants in Seattle, WA. Mislabeling rates identified by DNA sequencing are displayed (percent per category) as stacked bars. Percent per stack was calculated as follows = [(total mislabeled samples in a category)/(total samples of all categories)]*100%. Correctly labeled samples are also shown.

Of the 67 grocery store samples, DNA sequencing revealed 9 were mislabeled (Table 1). One of the grocery store samples substituted Atlantic salmon (*S*. *salar*) with steelhead trout (*O*. *mykiss*) (Table 1), which genetically groups closer to Pacific salmon species than Atlantic salmon [47, 48]. Of the 52 sushi restaurant samples, DNA sequencing revealed 12 were mislabeled (Table 2). Overall, we found 21 of 119 (18%) of salmon samples from grocery stores and sushi restaurants combined were mislabeled (Tables 1 and 2), indicating the mislabeling of salmon is still a problem in Seattle. There was no significant difference in the salmon mislabeling rates of 13.5% in Seattle grocery stores (9 of 67 samples mislabeled) vs 23.1% in sushi restaurants (12 of 54 samples mislabeled) (p = 0.641) (Fig 2). The overall 13.5% mislabeling rate in Seattle grocery stores included a 9% mislabeling rate of vendor-claimed wild Pacific salmon being substituted with another wild Pacific salmon species, 3% mislabeling rate of vendor-claimed farmed Atlantic salmon being substituted with wild Pacific salmon species, and a 1.5% mislabeling rate of vendor-claimed farmed Atlantic salmon being substituted with steelhead trout (Fig 2). The overall 23.1% mislabeling rate in Seattle sushi restaurants included a 19.2% mislabeling rate of vendor-claimed wild Pacific salmon being substituted with farmed Atlantic salmon and a 3.8% mislabeling rate of vendor-claimed wild Pacific salmon being substituted with another wild Pacific salmon species (Fig 2).

**Table 1:** Salmon substitutions in Seattle WA. Grocery stores.

District	Vendor Claimed Salmon species Identity	DNA Sequence Identity	Grocery Store Type	Substitution benefits the Customer or Seller?	Customer Gain (+) or Loss (-) Due to Mislabeling ($/pound)
7	Atlantic (*Salmo salar*)	Sockeye (*Onchorhyncus nerka*)	Independent	Customer	+ $3.10
6	Atlantic (*Salmo salar*)	Steelhead Trout (*Onchorhyncus mykiss*)	Local Chain	Seller	- $0.87
5	Atlantic (*Salmo salar*)	Sockeye (*Onchorhyncus nerka*)	Local Chain	Customer	+ $3.10
4	Coho (*Onchorhyncus kisutch*)	Sockeye (*Onchorhyncus nerka*)	Independent	Customer	+ $2.67
7	Sockeye (*Onchorhyncus nerka*)	Coho (*Onchorhyncus kisutch*)	National Chain	Seller	- $2.67
6	Sockeye (*Onchorhyncus nerka*)	Chinook* (*Onchorhyncus tshawytscha*)	National Chain	Customer	+ $9.21
3	Sockeye (*Onchorhyncus nerka*)	Coho (*Onchorhyncus kisutch*)	National Chain	Seller	- $2.67
2	Sockeye (*Onchorhyncus nerka*)	Coho (*Onchorhyncus kisutch*)	Local Chain	Seller	- $2.67
3	Chinook* (*Onchorhyncus tshawytscha*)	Coho (*Onchorhyncus kisutch*)	Independent	Seller	- $11.88

Verbal declarations were used to confirm fish identity by all vendors. The price per pound of each salmon species was determined by averaging the marketed prices from the grocery store samples collected in our study. Steelhead trout average pricing was determined by averaging the price per pound from five different grocery stores in Seattle as the pricing was not available from the store it was purchased from. * Chinook salmon was marketed as “king” salmon by Seattle vendors.

**Table 2:** Salmon substitutions in Seattle WA. Sushi restaurants.

District	Vendor Claimed Salmon species Identity	DNA Sequence Identity	Substitution benefits the Customer or Seller?	Customer Gain (+) or Loss (-) Due to Mislabeling ($/pound)
3	Chinook* (*Onchorhyncus tshawytscha*)	Atlantic (*Salmo salar*)	Seller	- $12.31
4	Chinook* (*Onchorhyncus tshawytscha*)	Atlantic (*Salmo salar*)	Seller	- $12.31
6	Coho (*Onchorhyncus kisutch*)	Atlantic (*Salmo salar*)	Seller	- $0.43
7	Chinook* (*Onchorhyncus tshawytscha*)	Atlantic (*Salmo salar*)	Seller	- $12.31
4	Chinook* (*Onchorhyncus tshawytscha*)	Coho (*Onchorhyncus kisutch*)	Seller	- $11.88
7	Chinook* (*Onchorhyncus tshawytscha*)	Atlantic (*Salmo salar*)	Seller	- $12.31
3	Chinook* (*Onchorhyncus tshawytscha*)	Atlantic (*Salmo salar*)	Seller	- $12.31
6	Coho (*Onchorhyncus kisutch*)	Atlantic (*Salmo salar*)	Seller	- $0.43
7	*Wild caught (unknown species)*	Atlantic (*Salmo salar*)	Seller	- $0.43
7	Sockeye (*Onchorhyncus nerka*)	Atlantic (*Salmo salar*)	Seller	- $5.10
1	Sockeye (*Onchorhyncus nerka*)	Coho (*Onchorhyncus kisutch*)	Seller	- $2.67
4	*Wild caught (unknown species)*	Atlantic (*Salmo salar*)	Seller	- $0.43

Verbal declarations were used to confirm fish identity by all vendors. The price per pound of each salmon species was determined by averaging the marketed prices from the grocery store samples collected in our study. When the vendor claimed it was “wild” salmon but did not know what kind of wild salmon it was, we conservatively priced it as coho salmon since it was the cheapest wild salmon in our analysis.* Chinook salmon was marketed as “king” salmon by Seattle vendors.

**Fig 3:**
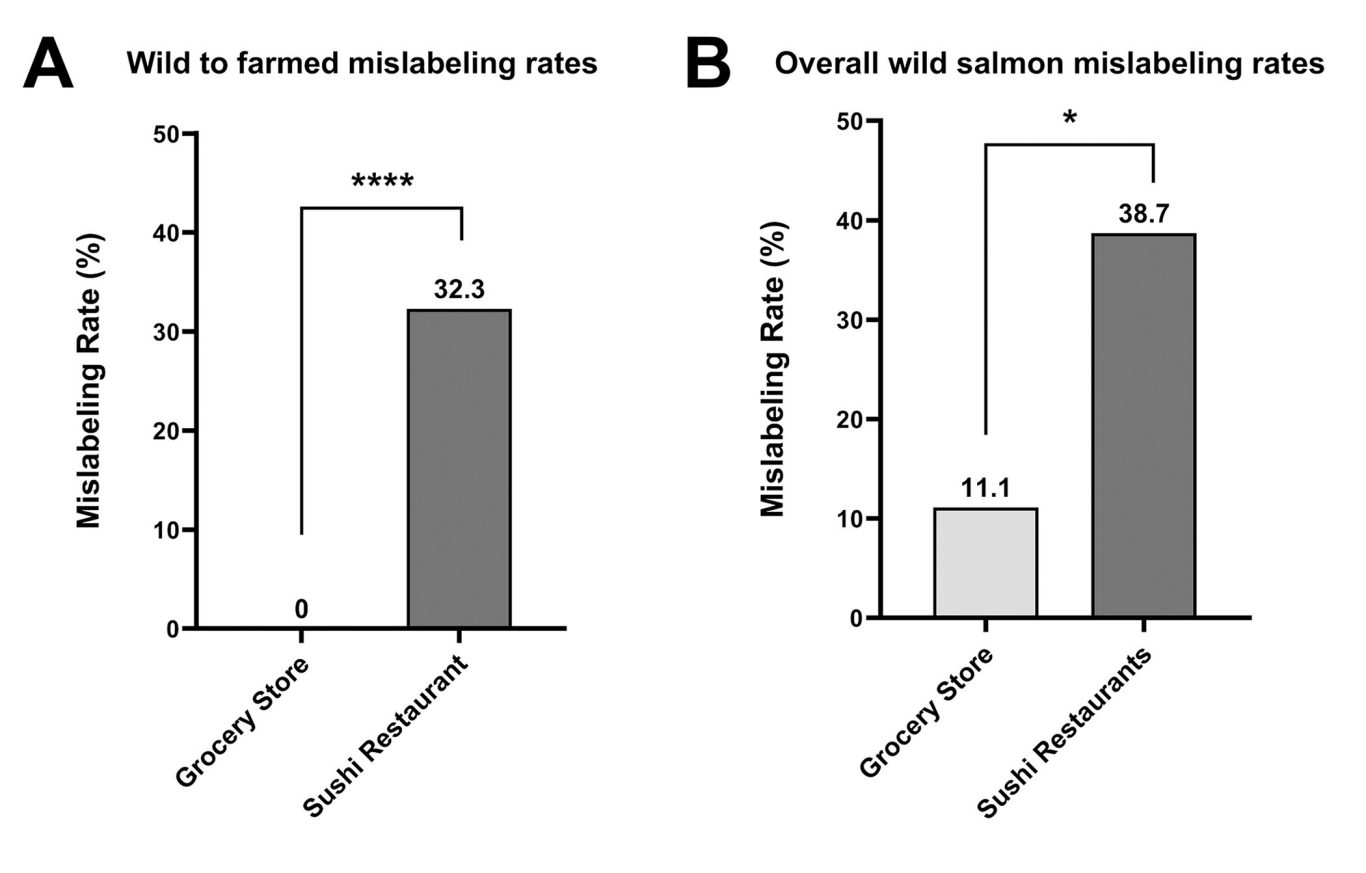
Wild salmon mislabeling rates in Seattle, WA. Wild salmon mislabeling rates were determined in Seattle grocery store samples (n = 54) vs sushi restaurant samples (n = 31). A) Wild to farmed salmon substitution rates were calculated as follows = [(total wild to farmed salmon mislabeled samples)/(total vendor claimed wild salmon samples)]*100%. B) Overall wild salmon substitution rates were calculated as follows = [(total wild salmon substituted with another wild or farmed salmon species)/(total vendor claimed wild salmon samples)]*100%. * p < 0.05; **** p < 0.0001.

Focused analysis of only vendor-claimed wild salmon samples revealed a significant difference in the substitution of wild salmon with farmed salmon, with sushi restaurants having an overall higher mislabeling rate of 32.3% (10 of 31 samples mislabeled) compared to 0% (0 of 52 samples mislabeled) in Seattle grocery stores (p < 0.0001) (Fig 3A). Additionally, we found a significant difference in the occurrences of wild salmon being substituted with another salmon species (wild salmon species or farmed), with sushi restaurants more likely to mislabel wild salmon at a rate of 38.7% (12 of 31 samples mislabeled) compared to 11.1% (6 of 54 samples mislabeled) in grocery store samples (p = 0.020) (Fig 3B).

### Customer gain or loss due to salmon mislabeling

**Fig 4:**
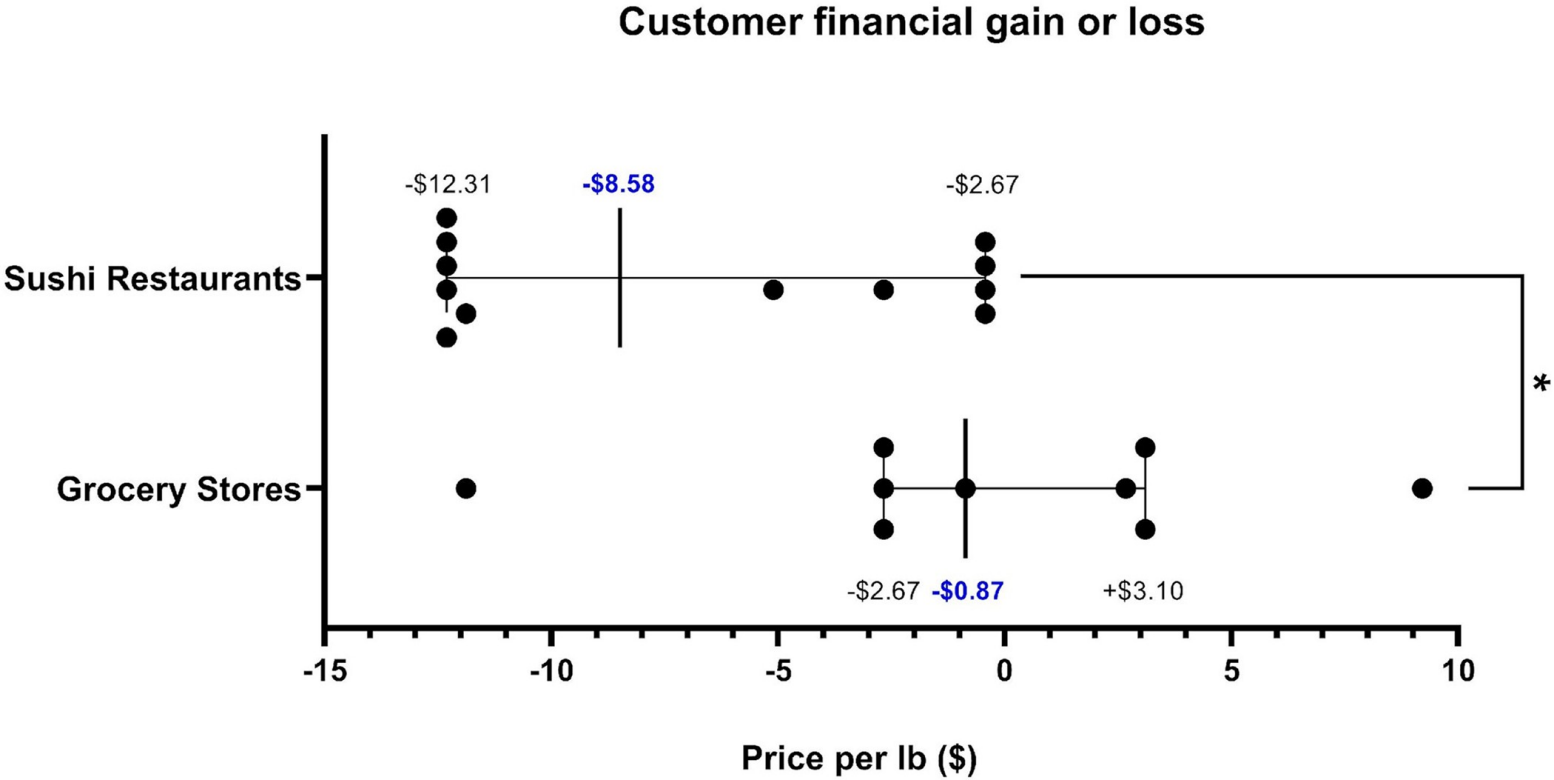
Customer gain vs loss analysis due to salmon substitutions in Seattle, WA. Grocery stores vs sushi restaurants. The median customer financial gain or loss ($/pound) of each mislabeled sample in Seattle grocery stores vs sushi restaurants are shown. The bars display the 95% CI of the median. * p < 0.05.

Any instance of salmon mislabeling results in consumer financial gain or loss, depending on if the substituted fish has a higher or a cheaper market price. For the Pacific salmon species, the average market price was $15.26/pound (or $6.92/kg) for coho, $17.93/pound (or $8.13/kg) for sockeye, and $27.14/pound (or $12.31/kg) for Chinook (S1 Fig). The average market pricing was $14.83/pound (or $6.73/kg) for farmed Atlantic salmon and $13.96/pound (or $6.33/kg) for steelhead (rainbow) trout (S1 Fig). While 60% of grocery store errors (Table 1) and 100% of sushi restaurant errors (Table 2) benefited the vendor, there was no significant difference between the two in how often errors benefited the vendor (p = 0.0816). However, there was a significant difference in the median gain or loss to the customer financially due to substitution events in grocery stores vs sushi restaurants (U_9,12_ = 84, p = 0.034), with a -$0.87 per pound loss per event (95% CI of the median = -$2.67/pound to +$3.10/pound) in grocery stores and -$8.58 per pound loss per event (95% CI of the median = -$12.31/pound to -$2.67/pound) in sushi restaurants (Fig 4). Furthermore, in grocery stores, we did not detect any significant economic loss to customers, as 95% CI’s overlapped $0.00. However, sushi customers experienced significant and systematic economic loss from salmon mislabeling.

## Discussion

Despite recent legislation (HB 1200) in Washington State making it illegal to knowingly sell seafood without identifying its common name, and making it unlawful for any retail salmon products to be sold without identifying it as wild-caught Pacific salmon or farm-raised Atlantic salmon, our study revealed Seattle is not immune to salmon fraud. Between 2022–2023, we found 18% of salmon samples from grocery stores and sushi restaurants combined were mislabeled. In comparison, a 20% salmon mislabeling rate was observed in Washington State between 2009–2011 [9] and a 2% salmon mislabeling rate was reported in Seattle in 2012 [17].

When comparing salmon mislabeling trends, it is important to consider which months the samples were collected. For example, studies show salmon mislabeling rates in the USA tend to increase in restaurants when salmon is acquired during the winter months (out-of-season) compared to late spring and summer months (in-season) [17, 18]. Salmon mislabeling rates in grocery stores, however, were found to remain similar regardless of the season [17, 18]. Furthermore, while the 2009–2011 Washington State study observed a high (20%) salmon mislabeling rate, half of the salmon samples were collected during the off-season winter months and half were collected during the early season spring months [9], when national salmon mislabeling trends are usually higher [18]. Moreover, the low (2%) salmon mislabeling rate in Seattle in 2012 could be attributed to various factors such as lower sample numbers (n = 60) compared to our study (n = 119), the fact that most samples were from grocery stores (which are often more reliable than restaurants), and sample collection only occurring when wild salmon was in season [17]. In comparison, even though the majority (82.3%) of our samples were collected during the fall months (end of wild salmon season) (S1 and S2 Tables) [52], we still observed a high (18%) overall salmon mislabeling rate. This was surprising as wild salmon was readily available in Seattle, indicating salmon mislabeling rates can still be high when wild salmon is in-season.

Regarding wild Pacific salmon substituted with farmed Atlantic salmon, we were encouraged to discover a 0% mislabeling rate in grocery stores compared to the 2% rate observed in 2009–2011 [9]. We are unaware of any other studies with similar sample sizes from any location that have demonstrated a complete lack of mislabeling fraud. In restaurants, we were concerned to find a 32.3% wild to farmed mislabeling rate in Seattle compared to the 24% rate in Washington State revealed 12 years ago [9]. We believe the current 0% wild to farmed salmon substitution rate in Seattle grocery stores is likely due to increased compliance with seafood identification laws in Washington State [43]. Additionally, the 0% rate may also be attributed to larger chain grocery stores committing to source their seafood from certified distributors which include seafood supply chain tracking [53, 54]. While it is unknown why the wild to farmed substitution rate in sushi restaurants did not go down after recent legislative changes, it is possible we observed higher mislabeling rates if their supply chain is less reliable compared to grocery stores. However, because the sources of farmed and wild salmon are completely distinct, mislabeling is likely not originating at the point of harvest (i.e., fishers and aquaculturists). This study reveals that customers should be wary of vendor-claimed salmon species when buying salmon, especially in sushi restaurants if the customer is expecting wild salmon. If a customer wants to buy wild salmon and avoid farmed salmon, Seattle grocery stores are a more reliable source.

Although grocery stores never supplied a customer with farmed salmon in place of wild salmon, we found 15.4% of vendor-claimed farmed salmon were actually wild sockeye salmon, meaning the customer benefited from the substitution by receiving a higher market-priced fish. Further, we did not detect significant economic loss to customers due to salmon mislabeling in grocery stores. As such, we found no evidence of systemic salmon mislabeling fraud within Seattle grocery stores. In contrast, all salmon substitutions in sushi restaurants harmed the customer financially by providing the customer with a less expensive salmon species, suggesting systemic mislabeling fraud within the sushi restaurant industry. Although we did not systematically categorize sushi restaurants based on average pricing, we observed fraudulent substitutions in both less-expensive and more-expensive sushi restaurants. For example, one sushi restaurant that only provided multi-course meals exceeding $100 a person, substituted Chinook salmon (i.e., the most expensive option) with farmed Atlantic salmon (i.e., the least expensive option). Importantly, while we were unable to determine where in the supply chain mislabeling occurred, many sushi restaurants could, conceivably, be victims of salmon mislabeling fraud if the mislabeling occurred higher in the supply chain.

As highlighted previously, the deceitful activity and subsequent jailing of one salmon distributor in Washington State led to an estimated $1.3 million in fraudulent profits in one year due to intentional substitution of vendor-claimed Chinook salmon with coho salmon [42]. When even lower-priced farmed fish are substituted in place of wild salmon, which is the most common substitution we observed, fraudulent profits would be even greater. More broadly, salmon fraud has the potential to result in the net loss of millions of dollars out of the pockets of Washington State customers each year. Additionally, fishers, seafood distributors, and retailers are negatively and financially affected if a cheaper priced mislabeled salmon product is sold on the market and outcompetes their honestly labeled and priced salmon [17]. Further, by mislabeling farmed salmon as wild, retailers are not only selling a lower market-priced product at inflated rates, they may also be selling consumers a product that consumers oppose on principle. For instance, survey data suggests that some consumers are willing to pay more for wild-caught fish than farmed fish because of environmental concerns related to aquaculture [55, 56]. Although salmon mislabeling may occur anywhere within the supply chain, reliable identification by consumers is often difficult. In grocery stores, whole fillets of salmon are easier to distinguish by both the vendor and customer as the skin and flesh of wild vs farmed salmon are visible. For example, wild salmon have a darker pinkish-orange color flesh compared to the grayish color of farmed salmon [55, 57, 58], but dietary supplements provided to farmed salmon may give them a pink appearance and therefore complicate identification [58]. On the other hand, sushi portions tend to be smaller, do not include skin, and are combined with other ingredients, complicating reliable identification for consumers. To help prevent mislabeling, the Washington Department of Fish and Wildlife distributes salmonid identification cards [59] that detail salmon species differences, such as Chinook contain dark mouths and black gums with spots on the upper and lower tail lobes, and coho have white mouths and almost white gum line with scattered spots on the upper tail lobe [59]. At the national level, the NOAA Fisheries Seafood Inspection Program provides a voluntary, fee-based inspection service to fishing boats, processing plants, and retailers to verify label accuracy and ensure compliance with all seafood regulations [60]. Additionally, the FDA periodically inspects and genetically tests seafood to ensure proper labeling and compliance with market names [60], as well as created a guide termed “The Seafood List’’ which contains acceptable market names for seafood products sold in the USA [61].

Furthermore, this study raises ecological and wild salmon sustainability concerns. Failure to label seafood correctly prevents accurate tracking of supply chains and complicates effective fishery management efforts. For instance, when lower market-priced, more common species, are mislabeled as higher market-priced, rarer species, fisheries managers may erroneously conclude that stocks which are actually depleted are persisting at sustainably harvestable densities [62]. Further, mislabeled seafood often involves substituting imported fish for fish that were labeled as domestically caught, and such substitutions often involve illegal, unreported, and unregulated (IUU) wild fish that come from overexploited populations [8, 63]. Many of the countries exporting wild-caught seafood to the USA have relatively weak environmental regulations and poorly managed stocks [64], thus mislabeling of wild-caught fish can directly contribute to the depletion of overfished IUU populations [65].

Mislabeling of farmed fish as wild-caught also masks broader ecological costs associated with aquaculture [44]. For instance, aquaculture feed may increase land use requirements when feed is produced terrestrially [66] and contribute to depletion of wild fish stocks when feed is produced from wild-harvested forage fish [67]. Additionally, fish farming has contributed to invasion of escaped farmed fish [45], transmission of disease and parasites [68–70], eutrophication from farm effluent [71, 72], and increased carbon emissions relative to wild harvested fish [72]. The use of antibiotics in aquaculture increases the incidence of antimicrobial resistance bacteria [73–75] and large scale antibiotic use during salmon farming can result in antibiotics leaking into the surrounding environment, altering and threatening the surrounding microbial communities, microbial diversity, and the ecosystem [76, 77]. The use of antibiotics in farmed salmon pens often raises ecological concerns for consumers and provides a principled incentive to buy wild salmon instead of farmed salmon [55, 56]. Further, when farmed salmon are fraudulently mislabeled as wild salmon, the country of origin of the farmed salmon remains undetermined, but aquaculture practices in countries exporting farmed fish to the USA often incur substantially greater environmental costs than domestic aquaculture [78].

## Conclusions

Salmon is the most frequently consumed finfish in the United States, and mislabeling events not only financially hurt the customer but can also negatively impact fishers, seafood distributors, retailers, and ecosystems. In this study, we identified a high level of salmon mislabeling in Seattle, WA. despite recent legislation which makes misidentification of salmon illegal. Seattle grocery stores tended to be more reliable compared to the sushi restaurant industry in properly labeling their salmon, especially when identifying it as wild vs farmed. Improper labeling of wild salmon, either as a different species of wild salmon or as farmed salmon, prevents accurate tracking of supply chains and complicates efforts to ensure that wild salmon originate from legal, well-regulated fisheries. Improper labeling of farmed salmon as wild salmon obscures negative environmental externalities often associated with aquaculture, and harms consumers by substituting a higher market-priced species with a lower market priced species. Taken together, it is important to continue developing and enforcing both federal and international policies that require accurate seafood labeling throughout the supply chain, from fisher to plate, and the use of ecologically sustainable fishing practices. It is also important to educate the public on the incidence of salmon fraud and its impact on salmon conservation.

## Supporting information

S1 Table Sushi restaurant sample information. (XLSX)

S2 Table Grocery store sample information. (XLSX)

S1 Data Sushi restaurant and grocery store sample DNA sequencing FASTA. (ZIP)

S1 Fig Average grocery store salmon prices in Seattle, WA. The mean dollar per pound for each salmon species was determined by averaging the grocery store market price each sample was acquired from. Error bars show +1.0 standard error of the mean.
(TIF)

## References

[1] Pauly D, Christensen V, Dalsgaard J, Froese R, Torres F. Fishing Down Marine Food Webs. Science. 1998;279: 860–863. doi: 10.1126/science.279.5352.860 9452385

[2] Pauly D, Zeller D. Catch reconstructions reveal that global marine fisheries catches are higher than reported and declining. Nat Commun. 2016;7: 10244. doi: 10.1038/ncomms10244 26784963 PMC4735634

[3] Worm B, Barbier EB, Beaumont N, Duffy JE, Folke C, Halpern BS, et al. Impacts of Biodiversity Loss on Ocean Ecosystem Services. Science. 2006;314: 787–790. doi: 10.1126/science.1132294 17082450

[4] Bell JD, Watson RA, Ye Y. Global fishing capacity and fishing effort from 1950 to 2012. Fish and Fisheries. 2017;18: 489–505. doi: 10.1111/faf.12187

[5] Palomares MLD, Pauly D. On the creeping increase of vessels’ fishing power. Ecology and Society. 2019;24. doi: 10.5751/ES-11136-240331

[6] Watson R, Pauly D. Systematic distortions in world fisheries catch trends. Nature. 2001;414: 534–536. doi: 10.1038/35107050 11734851

[7] Cawthorn D-M, Mariani S. Global trade statistics lack granularity to inform traceability and management of diverse and high-value fishes. Sci Rep. 2017;7: 12852. doi: 10.1038/s41598-017-12301-x 28993629 PMC5634443

[8] Kroetz K, Luque GM, Gephart JA, Jardine SL, Lee P, Chicojay Moore K, et al. Consequences of seafood mislabeling for marine populations and fisheries management. Proc Natl Acad Sci U S A. 2020;117: 30318–30323. doi: 10.1073/pnas.2003741117 33199620 PMC7720233

[9] Cline E. Marketplace substitution of Atlantic salmon for Pacific salmon in Washington State detected by DNA barcoding. Food Research International. 2012;45: 388–393. doi: 10.1016/j.foodres.2011.10.043

[10] Khaksar R, Carlson T, Schaffner DW, Ghorashi M, Best D, Jandhyala S, et al. Unmasking seafood mislabeling in U.S. markets: DNA barcoding as a unique technology for food authentication and quality control. Food Control. 2015;56: 71–76. doi: 10.1016/j.foodcont.2015.03.007

[11] Kitch CJ, Tabb AM, Marquis GE, Hellberg RS. Species substitution and mislabeling of ceviche, poke, and sushi dishes sold in Orange County, California. Food Control. 2023;146: 109525. doi: 10.1016/j.foodcont.2022.109525

[12] Muñoz-Colmenero M, Juanes F, Dopico E, Martinez JL, Garcia-Vazquez E. Economy matters: A study of mislabeling in salmon products from two regions, Alaska and Canada (Northwest of America) and Asturias (Northwest of Spain). Fisheries Research. 2017;195: 180–185. doi: 10.1016/j.fishres.2017.07.012

[13] Stern DB, Nallar EC, Rathod J, Crandall KA. DNA Barcoding analysis of seafood accuracy in Washington, D.C. restaurants. PeerJ. 2017;5: e3234. doi: 10.7717/peerj.3234 28462038 PMC5407275

[14] Wallstrom MA, Morris KA, Carlson LV, Marko PB. Seafood mislabeling in Honolulu, Hawai’i. Forensic Science International: Reports. 2020;2: 100154. doi: 10.1016/j.fsir.2020.100154

[15] Willette DA, Simmonds SE, Cheng SH, Esteves S, Kane TL, Nuetzel H, et al. Using DNA barcoding to track seafood mislabeling in Los Angeles restaurants. Conservation Biology. 2017;31: 1076–1085. doi: 10.1111/cobi.12888 28075039

[16] Wong EH-K, Hanner RH. DNA barcoding detects market substitution in North American seafood. Food Research International. 2008;41: 828–837. doi: 10.1016/j.foodres.2008.07.005

[17] Warner K, Timme W, Lowell B, Hirshfield M. Oceana Study Reveals Seafood Fraud Nationwide. Washington, DC: Oceana; 2013 Feb pp. 1–69. Available: https://oceana.org/wp-content/uploads/sites/18/National_Seafood_Fraud_Testing_Results_FINAL.pdf

[18] Warner K, Mustain P, Carolin C, Disla C, Kroner RG, Lowell B, et al. Oceana Reveals Mislabeling of America’s Favorite Fish: Salmon. Washington, DC: Oceana; 2015 Oct pp. 1–20. Available: https://usa.oceana.org/wp-content/uploads/sites/4/salmon_testing_report_finalupdated.pdf

[19] Armani A, Tinacci L, Lorenzetti R, Benvenuti A, Susini F, Gasperetti L, et al. Is raw better? A multiple DNA barcoding approach (full and mini) based on mitochondrial and nuclear markers reveals low rates of misdescription in sushi products sold on the Italian market. Food Control. 2017;79: 126–133. doi: 10.1016/j.foodcont.2017.03.030

[20] Chang C-H, Tsai M-L, Huang T-T, Wang Y-C. Authentication of fish species served in conveyor-belt sushi restaurants in Taiwan using DNA barcoding. Food Control. 2021;130: 108264. doi: 10.1016/j.foodcont.2021.108264

[21] Cundy ME, Santana-Garcon J, McLennan AG, Ayad ME, Bayer PE, Cooper M, et al. Seafood label quality and mislabelling rates hamper consumer choices for sustainability in Australia. Sci Rep. 2023;13: 10146. doi: 10.1038/s41598-023-37066-4 37537170 PMC10400555

[22] Han C, Dong S, Li L, Gao Q, Zhou Y. DNA Barcoding and Mini-DNA Barcoding Reveal Mislabeling of Salmonids in Different Distribution Channels in the Qingdao Area. Journal of Ocean University of China. 2021;20: 1537–1544. doi: 10.1007/s11802-021-4777-1

[23] Hu Y, Huang SY, Hanner R, Levin J, Lu X. Study of fish products in Metro Vancouver using DNA barcoding methods reveals fraudulent labeling. Food Control. 2018;94: 38–47. doi: 10.1016/j.foodcont.2018.06.023

[24] Liu B, Yang J-W, Liu B-S, Zhang N, Guo L, Guo H-Y, et al. Detection and identification of marine fish mislabeling in Guangzhou’s supermarkets and sushi restaurants using DNA barcoding. Journal of Food Science. 2022;87: 2440–2449. doi: 10.1111/1750-3841.16150 35438192

[25] Munguia-Vega A, Terrazas-Tapia R, Dominguez-Contreras JF, Reyna-Fabian M, Zapata-Morales P. DNA barcoding reveals global and local influences on patterns of mislabeling and substitution in the trade of fish in Mexico. PLOS ONE. 2022;17: e0265960. doi: 10.1371/journal.pone.0265960 35421106 PMC9009668

[26] Naaum A, Hanner R. Community engagement in seafood identification using DNA barcoding reveals market substitution in Canadian seafood. DNA Barcodes. 2015;3. doi: 10.1515/dna-2015-0009

[27] Prida V, Sepúlveda M, Quezada-Romegialli C, Harrod C, Gomez-Uchida D, Cid B, et al. Chilean Salmon Sushi: Genetics Reveals Product Mislabeling and a Lack of Reliable Information at the Point of Sale. Foods. 2020;9: 1699. doi: 10.3390/foods9111699 33228244 PMC7699462

[28] Tantillo G, Marchetti P, Mottola A, Terio V, Bottaro M, Bonerba E, et al. Occurrence of Mislabelling in Prepared Fishery Products in Southern Italy. Ital J Food Saf. 2015;4: 5358. doi: 10.4081/ijfs.2015.5358 27800410 PMC5076639

[29] Velez-Zuazo X, Alfaro-Shigueto J, Rosas-Puchuri U, Guidino C, Pasara-Polack A, Riveros JC, et al. High incidence of mislabeling and a hint of fraud in the *ceviche* and sushi business. Food Control. 2021;129: 108224. doi: 10.1016/j.foodcont.2021.108224

[30] Wang N, Xing R-R, Zhou M-Y, Sun R-X, Han J-X, Zhang J-K, et al. Application of DNA barcoding and metabarcoding for species identification in salmon products. Food Additives & Contaminants: Part A. 2021;38: 754–768. doi: 10.1080/19440049.2020.1869324 33783328

[31] Luque GM, Donlan CJ. The characterization of seafood mislabeling: A global meta-analysis. Biological Conservation. 2019;236: 556–570. doi: 10.1016/j.biocon.2019.04.006

[32] Ward RD, Zemlak TS, Innes BH, Last PR, Hebert PDN. DNA barcoding Australia’s fish species. Philos Trans R Soc Lond B Biol Sci. 2005;360: 1847–1857. doi: 10.1098/rstb.2005.1716 16214743 PMC1609232

[33] National Marine Fisheries Service. Fisheries of the United States, 2020. U.S. Department of Commerce, NOAA Current Fishery Statistics No. 2020. 2022 May. Available: https://www.fisheries.noaa.gov/national/sustainable-fisheries/fisheries-united-states

[34] Quinn TP, Losee JP. Diverse and changing use of the Salish Sea by Pacific salmon, trout, and char. Can J Fish Aquat Sci. 2022;79: 1003–1021. doi: 10.1139/cjfas-2021-0162

[35] Pandey R, Asche F, Misund B, Nygaard R, Adewumi OM, Straume H-M, et al. Production growth, company size, and concentration: The case of salmon. Aquaculture. 2023;577: 739972. doi: 10.1016/j.aquaculture.2023.739972

[36] Jenkins D. Atlantic Salmon, Endangered Species, and the Failure of Environmental Policies. Comparative Studies in Society and History. 2003;45: 843–872. doi: 10.1017/S0010417503000379

[37] NOAA Fisheries. Atlantic Salmon: Aquaculture. [cited 13 Sep 2024]. Available: https://www.fisheries.noaa.gov/species/atlantic-salmon/aquaculture

[38] Ritchie M, Lepofsky D, Formosa S, Porcic M, Edinborough K. Beyond culture history: Coast Salish settlement patterning and demography in the Fraser Valley, BC. Journal of Anthropological Archaeology. 2016;43: 140–154. doi: 10.1016/j.jaa.2016.06.002

[39] WADOE. Fish Consumption Rates. Technical Support Document: A Review of Data and Information about Fish Consumption in Washington. Version 2.0. Final. Olympia, WA: Department of Ecology, State of Washington; 2013 Jan. Report No.: 12–09–058. Available: https://apps.ecology.wa.gov/publications/documents/1209058.pdf

[40] NOAA Fisheries. Fisheries of the United States. In: NOAA [Internet]. 4 Apr 2024 [cited 13 Sep 2024]. Available: https://www.fisheries.noaa.gov/national/sustainable-fisheries/fisheries-united-states

[41] Governor’s Salmon Recovery Office. 2022 State of Salmon in Watersheds Executive Summary. 2022. Available: https://stateofsalmon.wa.gov/wp-content/uploads/2023/02/SOS-ExecSummary-2022.pdf

[42] U.S. Attorney’s Office—U.S. Department of Justice. Fish Processor sentenced to prison for selling falsely labeled salmon. 1 Apr 2011 [cited 23 Apr 2024]. Available: https://www.justice.gov/archive/usao/waw/press/2011/apr/jay.html

[43] State of Washington. An act relating to labeling of seafood. SUBSTITUTE HOUSE BILL 1200 May 20, 2013 p. 18. Available: https://apps.leg.wa.gov/documents/billdocs/2013-14/Pdf/Bills/House%20Passed%20Legislature/1200-S.PL.pdf

[44] Diana JS. Aquaculture Production and Biodiversity Conservation. BioScience. 2009;59: 27–38. doi: 10.1525/bio.2009.59.1.7

[45] Clark D, Lee K, Murphy K, Windrope A. 2017 Cypress Island Atlantic Salmon Net Pen Failure: An Investigation and Review. Department of Natural Resources. Olympia, WA.; 2017. Available: https://www.dnr.wa.gov/sites/default/files/publications/aqr_cypress_investigation_report.pdf?vdqi7rk

[46] Franz Hilary (Commission of Public Lands). Commissioner’s Order on Commercial Finfish Net Pen Aquaculture. 2022–11 Nov 17, 2022. Available: https://www.dnr.wa.gov/publications/em_commissioners_order_net_pens.pdf

[47] Korzow Richter K, McGrath K, Masson-MacLean E, Hickinbotham S, Tedder A, Britton K, et al. What’s the catch? Archaeological application of rapid collagen-based species identification for Pacific Salmon. Journal of Archaeological Science. 2020;116: 105116. doi: 10.1016/j.jas.2020.105116

[48] Lecaudey LA, Schliewen UK, Osinov AG, Taylor EB, Bernatchez L, Weiss SJ. Inferring phylogenetic structure, hybridization and divergence times within Salmoninae (Teleostei: Salmonidae) using RAD-sequencing. Molecular Phylogenetics and Evolution. 2018;124: 82–99. doi: 10.1016/j.ympev.2018.02.022 29477383

[49] Adibah AB, Syazwan S, Haniza Hanim MZ, Badrul Munir MZ, Intan Faraha AG, Siti Azizah MN. Evaluation of DNA barcoding to facilitate the authentication of processed fish products in the seafood industry. LWT. 2020;129: 109585. doi: 10.1016/j.lwt.2020.109585

[50] Rasmussen RS, Morrissey MT, Hebert PDN. DNA Barcoding of Commercially Important Salmon and Trout Species (Oncorhynchus and Salmo) from North America. J Agric Food Chem. 2009;57: 8379–8385. doi: 10.1021/jf901618z 19705801

[51] Zar JH. Biostatistical analysis. 5th ed. Upper Saddle River, N.J: Prentice-Hall/Pearson; 2010.

[52] Washington Department of Fish and Wildlife. Washington Sport Fishing Rules: Effective July 1, 2024-June 30, 2025. Olympia, WA; 2024. Available: https://www.eregulations.com/washington/fishing/

[53] Kroger Seafood Sustainability Report 2009–2020. Available: https://www.thekrogerco.com/wp-content/uploads/2021/07/Kroger-2020-Seafood-Sustainability-Report.pdf

[54] 2023 Environmental Social Governance Report. Albertsons Companies; Available: https://s29.q4cdn.com/239956855/files/our_impact/sustainability_doc/albcdacsiv199540_aci_23_esg-report-1-2.pdf

[55] Davidson K, Pan M, Hu W, Poerwanto D. Consumers’ Willingness to Pay for Aquaculture Fish Products Vs. Wild-Caught Seafood–a Case Study in Hawaii. Aquaculture Economics & Management. 2012;16: 136–154. doi: 10.1080/13657305.2012.678554

[56] Roheim CA, Sudhakaran PO, Durham CA. Certification of shrimp and salmon for best aquaculture practices: assessing consumer preferences in rhode island. Aquaculture Economics & Management. 2012;16: 266–286. doi: 10.1080/13657305.2012.713075

[57] Alfnes F, Guttormsen AG, Steine G, Kolstad K. Consumers’ Willingness to Pay for the Color of Salmon: A Choice Experiment with Real Economic Incentives. American Journal of Agricultural Economics. 2006;88: 1050–1061. doi: 10.1111/j.1467-8276.2006.00915.x

[58] Buttle L, Crampton V, Williams P. The effect of feed pigment type on flesh pigment deposition and colour in farmed Atlantic salmon, Salmo salar L. Aquaculture Research. 2001;32: 103–111. doi: 10.1046/j.1365-2109.2001.00536.x

[59] The Trout and Salmon Identification Guide. Washington Department of Fish and Wildlife; 1995 Jun. Available: https://wdfw.wa.gov/sites/default/files/publications/00303/wdfw00303.pdf

[60] Fisheries N. Seafood Fraud | NOAA Fisheries. In: NOAA [Internet]. 21 Feb 2024 [cited 28 Apr 2024]. Available: https://www.fisheries.noaa.gov/national/sustainable-seafood/seafood-fraud

[61] The Seafood List. [cited 28 Apr 2024]. Available: https://www.cfsanappsexternal.fda.gov/scripts/fdcc/?set=SeafoodList

[62] Marko PB, Lee SC, Rice AM, Gramling JM, Fitzhenry TM, McAlister JS, et al. Mislabelling of a depleted reef fish. Nature. 2004;430: 309–310. doi: 10.1038/430309b 15254528

[63] Helyar SJ, Lloyd H ap D, Bruyn M de, Leake J, Bennett N, Carvalho GR. Fish Product Mislabelling: Failings of Traceability in the Production Chain and Implications for Illegal, Unreported and Unregulated (IUU) Fishing. PLOS ONE. 2014;9: e98691. doi: 10.1371/journal.pone.0098691 24921655 PMC4055496

[64] Smith MD, Roheim CA, Crowder LB, Halpern BS, Turnipseed M, Anderson JL, et al. Sustainability and Global Seafood. Science. 2010;327: 784–786. doi: 10.1126/science.1185345 20150469

[65] Jacquet JL, Pauly D. Trade secrets: Renaming and mislabeling of seafood. Marine Policy. 2008;32: 309–318. doi: 10.1016/j.marpol.2007.06.007

[66] Froehlich HE, Runge CA, Gentry RR, Gaines SD, Halpern BS. Comparative terrestrial feed and land use of an aquaculture-dominant world. Proc Natl Acad Sci USA. 2018;115: 5295–5300. doi: 10.1073/pnas.1801692115 29712823 PMC5960322

[67] Naylor RL, Goldburg RJ, Primavera JH, Kautsky N, Beveridge MCM, Clay J, et al. Effect of aquaculture on world fish supplies. Nature. 2000;405: 1017–1024. doi: 10.1038/35016500 10890435

[68] Krkošek M, Ford JS, Morton A, Lele S, Myers RA, Lewis MA. Declining Wild Salmon Populations in Relation to Parasites from Farm Salmon. Science. 2007;318: 1772–1775. doi: 10.1126/science.1148744 18079401

[69] Torrissen O, Jones S, Asche F, Guttormsen A, Skilbrei OT, Nilsen F, et al. Salmon lice–impact on wild salmonids and salmon aquaculture. J Fish Dis. 2013;36: 171–194. doi: 10.1111/jfd.12061 23311858 PMC3675643

[70] Mordecai GJ, Miller KM, Bass AL, Bateman AW, Teffer AK, Caleta JM, et al. Aquaculture mediates global transmission of a viral pathogen to wild salmon. Sci Adv. 2021;7: eabe2592. doi: 10.1126/sciadv.abe2592 34039598 PMC8153721

[71] Saikku L, Asmala E. Eutrophication in the Baltic Sea. Journal of Industrial Ecology. 2010;14: 482–495. doi: 10.1111/j.1530-9290.2010.00221.x

[72] Ziegler F, Hilborn R. Fished or farmed: Life cycle impacts of salmon consumer decisions and opportunities for reducing impacts. Science of The Total Environment. 2023;854: 158591. doi: 10.1016/j.scitotenv.2022.158591 36089015

[73] Higuera-Llantén S, Vásquez-Ponce F, Barrientos-Espinoza B, Mardones FO, Marshall SH, Olivares-Pacheco J. Extended antibiotic treatment in salmon farms select multiresistant gut bacteria with a high prevalence of antibiotic resistance genes. PLoS One. 2018;13: e0203641. doi: 10.1371/journal.pone.0203641 30204782 PMC6133359

[74] Lozano-Muñoz I, Wacyk J, Kretschmer C, Vásquez-Martínez Y, Martin MC-S. Antimicrobial resistance in Chilean marine-farmed salmon: Improving food safety through One Health. One Health. 2021;12: 100219. doi: 10.1016/j.onehlt.2021.100219 33553565 PMC7856317

[75] Okocha RC, Olatoye IO, Adedeji OB. Food safety impacts of antimicrobial use and their residues in aquaculture. Public Health Rev. 2018;39: 21. doi: 10.1186/s40985-018-0099-2 30094087 PMC6081861

[76] Cabello FC, Godfrey HP, Buschmann AH, Dölz HJ. Aquaculture as yet another environmental gateway to the development and globalisation of antimicrobial resistance. The Lancet Infectious Diseases. 2016;16: e127–e133. doi: 10.1016/S1473-3099(16)00100-6 27083976

[77] Watts JEM, Schreier HJ, Lanska L, Hale MS. The Rising Tide of Antimicrobial Resistance in Aquaculture: Sources, Sinks and Solutions. Marine Drugs. 2017;15: 158. doi: 10.3390/md15060158 28587172 PMC5484108

[78] Kite-Powell HL, Rubino MC, Morehead B. The Future of U.S. Seafood Supply. Aquaculture Economics & Management. 2013;17: 228–250. doi: 10.1080/13657305.2013.812691

