# Supplementary figures and images for: Fishy business in Seattle: Salmon mislabeling fraud in sushi restaurants vs grocery stores
Source: PLoS One. 2024 Nov 6;19(11):e0311522. doi: 10.1371/journal.pone.0311522 (PMC11540171; doi:10.1371/journal.pone.0311522)

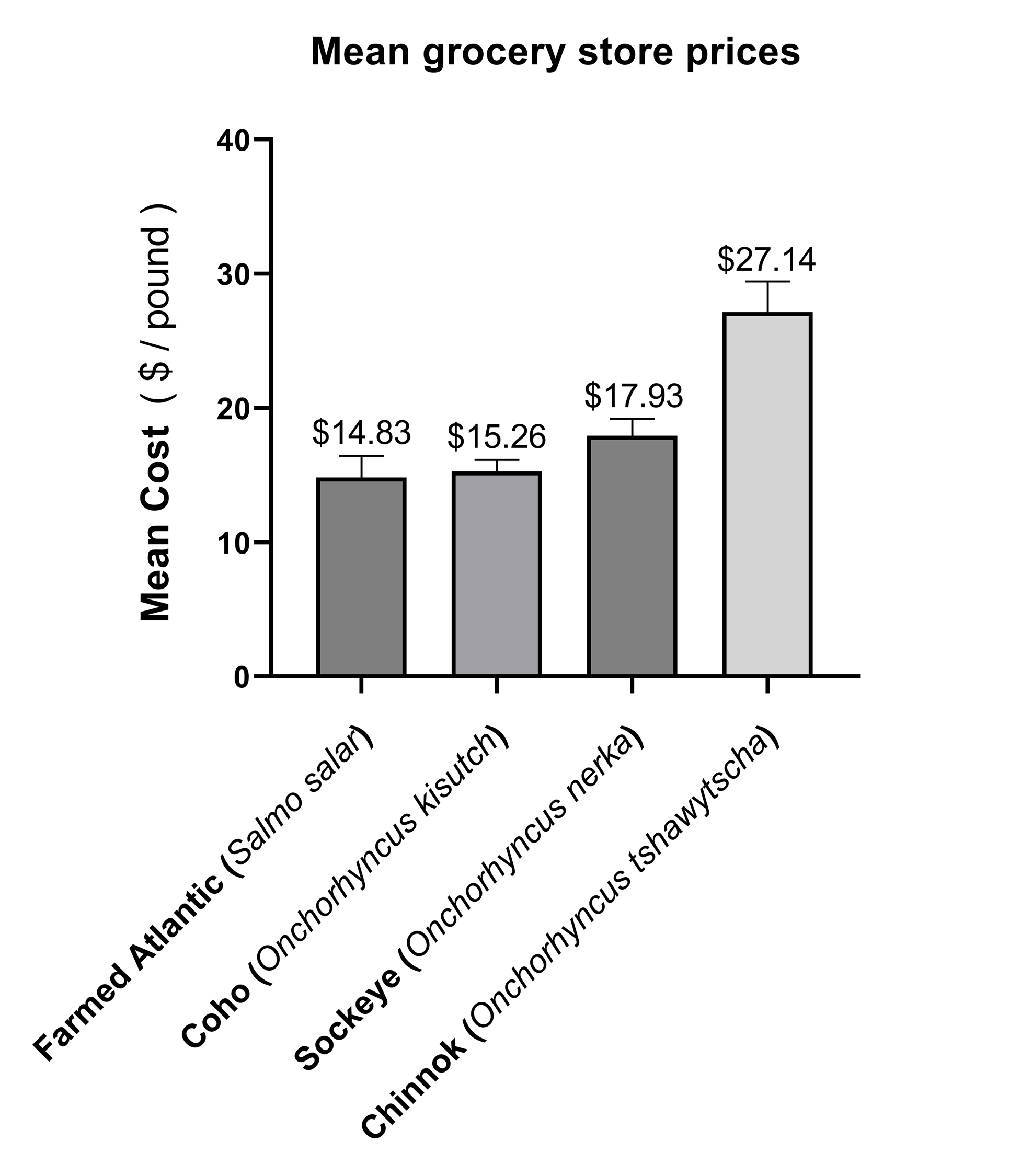

Supplement: S1 Fig — The mean dollar per pound for each salmon species was determined by averaging the grocery store market price each sample was acquired from. Error bars show +1.0 standard error of the mean. (TIF) [file pone.0311522.s004.tif]
