# Supplementary material for: Fishy business in Seattle: Salmon mislabeling fraud in sushi restaurants vs grocery stores
Source: PLoS One. 2024 Nov 6;19(11):e0311522. doi: 10.1371/journal.pone.0311522 (PMC11540171; doi:10.1371/journal.pone.0311522)
Supplement: S1 Data — (ZIP) [file pone.0311522.s003.zip › S1 Data/Sushi Restaurant FASTA files/S49.doc]

>S49

NNNNNNNNNNNNNNNNNNGNGCNNGNNCCGGATAGTCGGCACCGCCCTAA

GTCTCTTGATTCGAGCAGAACTCAGCCAGCCTGGCGCCCTTCTGGGAGAT

GACCAAATTTATAACGTAATTGTTACAGCCCATGCCTTCGTCATAATTTT

CTTTATAGTCATACCGATTATGATCGGCGGCTTTGGAAACTGATTAATTC

CTCTTATAATCGGGGCCCCCGACATAGCATTCCCCCGAATGAATAACATA

AGTTTTTGACTTCTCCCTCCCTCCTTTCTTCTCCTCCTGGCCTCATCTGG

AGTTGAAGCCGGCGCTGGCACCGGATGAACAGTCTACCCCCCTCTAGCAG

GTAATCTTGCCCACGCAGGAGCTTCCGTTGACTTAACTATTTTTTCCCTC

CATTTGGCTGGTATTTCTTCAATTCTTGGGGCCATTAATTTTATTACAAC

CATTATTAATATAAAACCCCCAGCTATCTCTCAGTATCAAACCCCACTTT

TTGTTTGAGCTGTATTAGTCACTGCCGTCCTTTTGTTACTCTCCCTCCCT

GTTCTAGCAGCAGGCATTACCATACTACTTACAGACCGAAATCTAAATAC

CACTTTCTTTGACCCGGCAGGCGGANGAGACCCAATCTTGTACCAACATC

TCTTTTGATTCTTTGGCCNNNNNNAAGTCTAANNNN
